# New spin coated multilayer lactate biosensor for acidosis monitoring in continuous flow assisted with an electrochemical pH probe

**DOI:** 10.1007/s00604-024-06602-y

**Published:** 2024-08-09

**Authors:** Juan José García-Guzmán, José Manuel Jiménez Heras, David López-Iglesias, Rafael Jesús González-Álvarez, Laura Cubillana-Aguilera, Carmen González Macías, Juan Jesús Fernández Alba, José María Palacios-Santander

**Affiliations:** 1 Instituto de Investigación e Innovación Biomédica de Cádiz (INiBICA), Hospital Universitario ‘Puerta del Mar’, Universidad de Cádiz, 11009 Cádiz, Spain; 2 Institute of Research on Electron Microscopy and Materials (IMEYMAT), Department of Analytical Chemistry, Faculty of Sciences, Campus de Excelencia Internacional del Mar (CEIMAR), Universidad de Cádiz, República Saharaui, S/N. 11510 Puerto Real, Cádiz, Spain; 3 Departamento de Obstetricia y Ginecología, Hospital Universitario de Puerto Real, 11510 Puerto Real, Cádiz, Spain

**Keywords:** Lactate amperometric biosensor, Diffusion limiting membrane, Spin coating procedure, Continuous monitoring, Healthcare assessment

## Abstract

A LOx-based electrochemical biosensor for high-level lactate determination was developed. For the construction of the biosensor, chitosan and Nafion layers were integrated by using a spin coating procedure, leading to less porous surfaces in comparison with those recorded after a drop casting procedure. The analytical performance of the resulting biosensor for lactate determination was evaluated in batch and flow regime, displaying satisfactory results in both modes ranging from 0.5 to 20 mM concentration range for assessing the lactic acidosis. Finally, the lactate levels in raw serum samples were estimated using the biosensor developed and verified with a blood gas analyzer. Based on these results, the biosensor developed is promising for its use in healthcare environment, after its proper miniaturization. A pH probe based on common polyaniline-based electrochemical sensor was also developed to assist the biosensor for the lactic acidosis monitoring, leading to excellent results in stock solutions ranging from 6.0 to 8.0 mM and raw plasma samples. The results were confirmed by using two different approaches, blood gas analyzer and pH-meter. Consequently, the lactic acidosis monitoring could be achieved in continuous flow regime using both (bio)sensors.

**Supplementary Information:**

The online version contains supplementary material available at 10.1007/s00604-024-06602-y.

## Introduction

Lactate (LA) is gaining attention in scientific community and, surprisingly, not only in the classic clinic/healthcare environment but also in the agrifood field [1, 2]. In the former, LA in biofluids (i.e., blood and sweat) results from the balance between production and uptake of lactate in human body during metabolism. During exercise, analyte levels are increased to fulfil the high requirements of the body. Therefore, a high level of LA is expected during intensive training, but it can be also observed due to sickness related with impaired metabolism or excessive production. Long periods of lactate accumulation led to the so-called lactic acidosis, which could cause several problems, and thus, LA monitoring can be extremely valuable in the medical frame. LA is also very useful in food control, providing relevant information regarding the decaying grade of the foodstuff and the stress monitoring of living species [3]. Based on its relevance, a great number of researchers are devoting their efforts to develop continuous monitoring approaches for LA determination by using statistical tools [4, 5]. In this regard, the advantages of electrochemical devices are well known, such as simplicity, quickness, high sensitivity, cost effectiveness, no sample preparation required, portability, and the possibility of in situ and online analysis by using minimally invasive procedures [6]. Interestingly, some non-enzymatic approaches to determine lactate have been developed [7]. In spite of the non-enzymatic possibilities, enzyme-based biosensors are still the main alternative to explore, considering their high selectivity and catalytic properties. Their modification with some nanomaterials, such as reduced graphene oxide and carbon nanotubes, led to an enhancement of the analytical performance due to their high electrocatalytic effect, electron transfer activity, chemical stability, and low cost [8]. Gold nanoparticles (AuNPs) modification has been exploited as modifier, improving the catalytic activity and retaining the native conformation at the same time [9]. Other relevant aspect is the employment of Prussian blue (PB) as a redox mediator to decrease the working potential and, consequently, diminish interference issues [10]. Despite their multiple advantages, enzyme-based biosensors have also inherited one important drawback, enzymatic saturation. Remarkably, high concentration level of lactate is usually found in the most significant scenarios such as sickness or high effort situation in people, being possible to reach 25 and 50 mM of lactate in human blood and sweat, respectively [11]. This is why there is a clear need to expand as much as possible the linear range of lactate biosensors to pursue in situ application of the sensors. Diffusion limiting layers as coverages of biosensor surfaces are reported as a suitable alternative, since the transport of the analyte within the enzymatic layer was allowed gradually. One of the first approaches based on the employment of a polyurethane membrane as diffusion limiting layer was published by Burmeister et al. in 2005 [12]. This concept has also been very recently exploited aiming for the benefits of an expanded linear range as well as its other advantages (e.g., reduced interference influence) [13]. In this scenario, the deposition of different biopolymers, such as chitosan and polyurethane, by using drop casting was reported in the literature [12, 14, 15]. The deposition of Nafion® layer has been also reported as anionic barrier by other authors [16]. The employment of spin coating procedures for the deposition of multipolymeric layers has also been explored, since thicker layers were obtained by using spin coating procedures in comparison with the traditional drop casting ones [17]. Other relevant aspect to expand the linear range could be the deposition of a thick layer of Prussian blue, mainly attributed to the higher availability of the catalytic places [9, 18].

The lactic acidosis also led to lower pH in the organism, which is traduced into alterations in the acid–base homeostasis and massive damage. In fact, lactic acidosis (pH < 7.35) in association with higher levels of lactate (> 5 mM) carries a mortality of 80% in critical patients [19]. For this reason, the pH assessment is also valuable for lactic acidosis diagnosis. Potentiometry and ion selective electrodes (ISEs) constitute a classic approach to face this challenge. Briefly, these devices are based on ion selective membranes (ISMs), permeable only to a certain ion (i.e., H^+^), in combination with an ion-to electron transducer (e.g., functionalized carbon nanotubes) [20]. On the other hand, all-solid-state ISEs are a popular research topic, and hence, the employment of conducting polymers are still exploring [21]. In this regard, PANI-based sensors are very selective to H^+^, and the phase transition between emeraldine salt and emeraldine base provides a convenient manner to determine the pH [22].

**Fig. 1:**
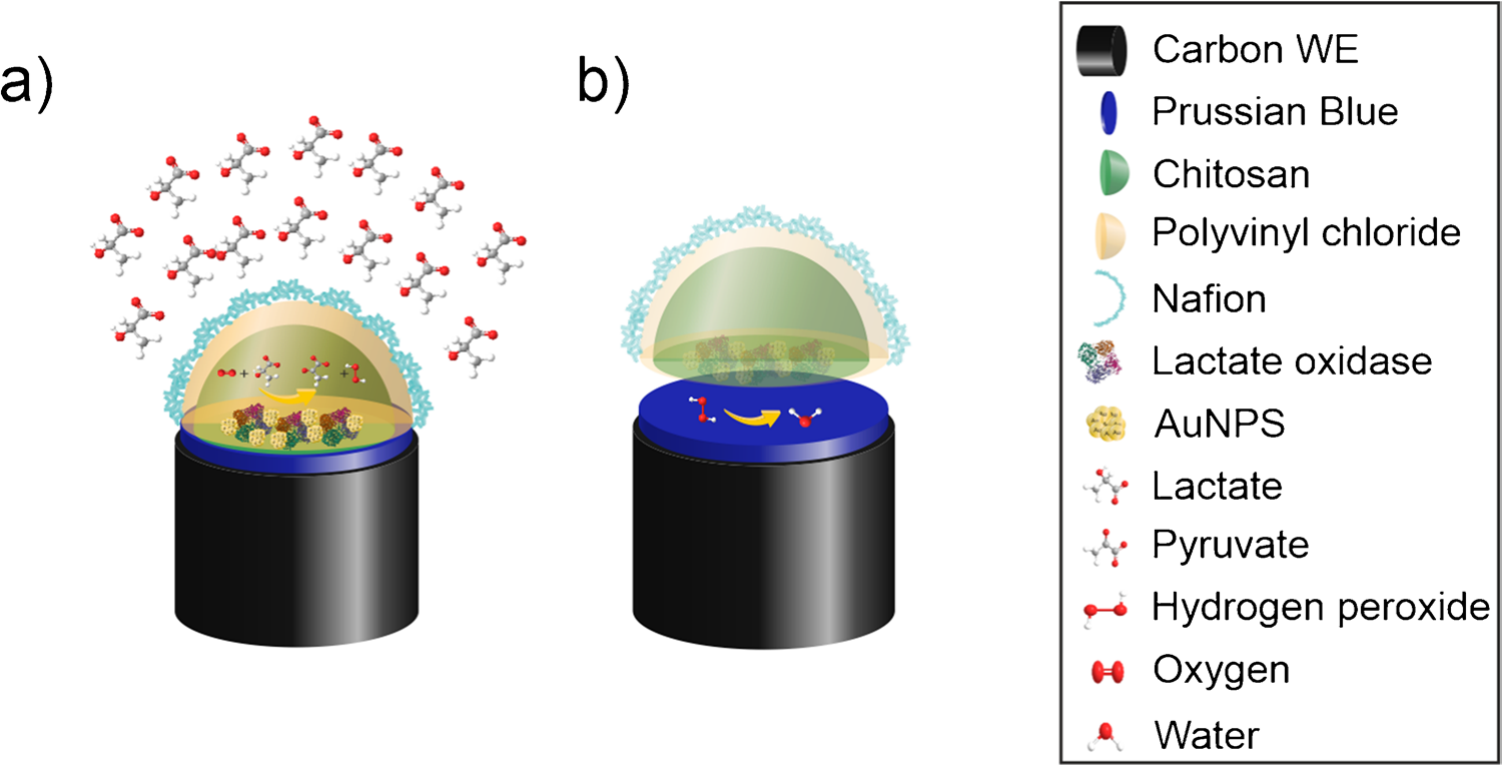
Conceptual scheme of the proposed mechanism of lactate detection with the SCPC-PB-LOxAuNPs-Chit-PVC-Nafion biosensor. **a** Oxygen and lactate reaching the enzymatic layer through the diffusion limiting layer and resulting into pyruvate and oxygen peroxide. **b** Later transformation of hydrogen peroxide into water due to the catalytic effect of PB layer

In this work, we herein report a lactate biosensor based on a screen-printed carbon electrode with Prussian blue as redox mediator, lactate oxidase with AuNPs as biological recognition element and a multipolymeric membrane as diffusion limiting layer (SCPC-PB-LOxAuNPs-Chit-PVC-Nafion) (Fig. 1).

The polymeric membranes based on chitosan, polyvinyl chloride (PVC), and Nafion® have been partially prepared by using a spin coating procedure. The developed biosensor possesses some advantages for the lactate determination in comparison with other routine analytical techniques, such as high-pressure liquid chromatography (HPLC). Low-time consuming analyses (ca. few seconds) were accomplished by using the developed biosensor, in contrast with higher analysis time required for HPLC technique. Furthermore, electrochemical analysis of human serum could be performed without applying any pretreatment, while sample preparation is often required before performing HPLC assays. On the other hand, online measurements could be accomplished with the biosensor, since it could be miniaturized and coupled with a sample collector for the LA continuous monitoring, allowing its application in a real scenario. Although some LA biosensors employing flow injection analysis were already reported [23], the biosensor developed in this work is proposed to be used in clinical environment for lactic acidosis diagnosis after its further miniaturization.

In parallel, a pH probe based on electrodeposited polyaniline (PANI) has been also developed to assist the lactate sensor and aid a better diagnosis of lactic acidosis. The electrochemical probe was also tested for the pH determination in human plasma samples, leading to successful results in comparison with those obtained with the gold technique.

## Materials and methods

### Reagents and solutions

Lactate oxidase enzyme (LOx) from *Aerococcus viridans*, lyophilized powder (> 100 U mg^−1^); perfluorinated resin solution containing 5% wt Nafion® in ethanol; polyvinylbutyral (PVB); polyurethane, selectophore grade, high molecular weight; polyvinyl chloride; medium molecular weight chitosan; potassium hexacyanoferrate (III) (≥ 99%); L-( +)-lactic acid solution in water (≥ 85%); iron (III) chloride (97%); potassium tetrachloroaurate (III), (99.995% trace metals basis); and tetrahydrofuran, selectophore grade (99%) were obtained from Aldrich (Germany). Sodium citrate tribasic hydrate puriss (99%) and aniline (99.5%) were obtained from Riedel-de Haën–Honeywell (Germany). Potassium di-hydrogen phosphate (99.5%), di-potassium hydrogen phosphate anhydrous (99%), potassium hydroxide (85%), and glacial acetic acid were purchased from Panreac (Spain). Hydrochloric acid (35–38%) was from Labkem (Spain). All the solutions were made by using nanopure water (18 MΩ·cm of resistivity) from Wasser Lab Ultramatic Plus (Type I) system (Barbatáin, Navarra, Spain).

### Apparatus and instrumental analyses

The electrochemical measurements were carried out using an Autolab potentiostat/galvanostat 302N (Ecochemie, The Netherlands). Processing data was made using GPES (General Purpose Electrochemical System) software. Screen-printed carbon electrodes (SCPC) (DRP-11L) from Metrohm (Switzerland) were employed as the three electrodes system. The AFM images were obtained using a Dimension Icon microscope (Bruker) operating in Peak Force Tapping mode using ScanAsyst-Air probes (stiffness 0.2 − 0.8 N/m, frequency ∼80 kHz). Spin coating procedure was carried out by using a homemade rotor instrument assisted by 3D tools tailored for this purpose. The additive manufacturing was accomplished by using a PRUSA MINI printer and polylactic acid (PLA) filament both from Prusa Research (Czech Republic). Flow analysis was assisted by a flow cell for screen-printed electrodes 26 DRP-FLWCL from Metrohm (Switzerland), a Minipuls 2 from Gilson (USA) peristaltic pump, and a six-way valve from Omnifit. The gold reference technique to validate the sensors was a GEM Premier 4000 analyzer, which use multisample commercial cartridges.

### Real sample obtention

Blood real samples were obtained from nine volunteers in the University Hospital of Puerto Real (Cádiz, Spain); some of them subjected to moderate exercise before the blood collection. After performing the extraction procedure, blood was transferred to a special tube and centrifuged at 3500 rpm for 5 min to obtain the serum. Afterwards, the supernatant was transferred to Eppendorf tubes and stored in the freezer. Plasma samples were obtained from blood as well belonging to the same volunteers but using appropriate tubes for collecting. They were centrifuged at the same speed for 10 min to obtain the plasma. After centrifugation, the supernatant was transferred to Eppendorf tubes for their in situ analysis.

### (Bio)sensor preparation procedure

**Fig. 2:**
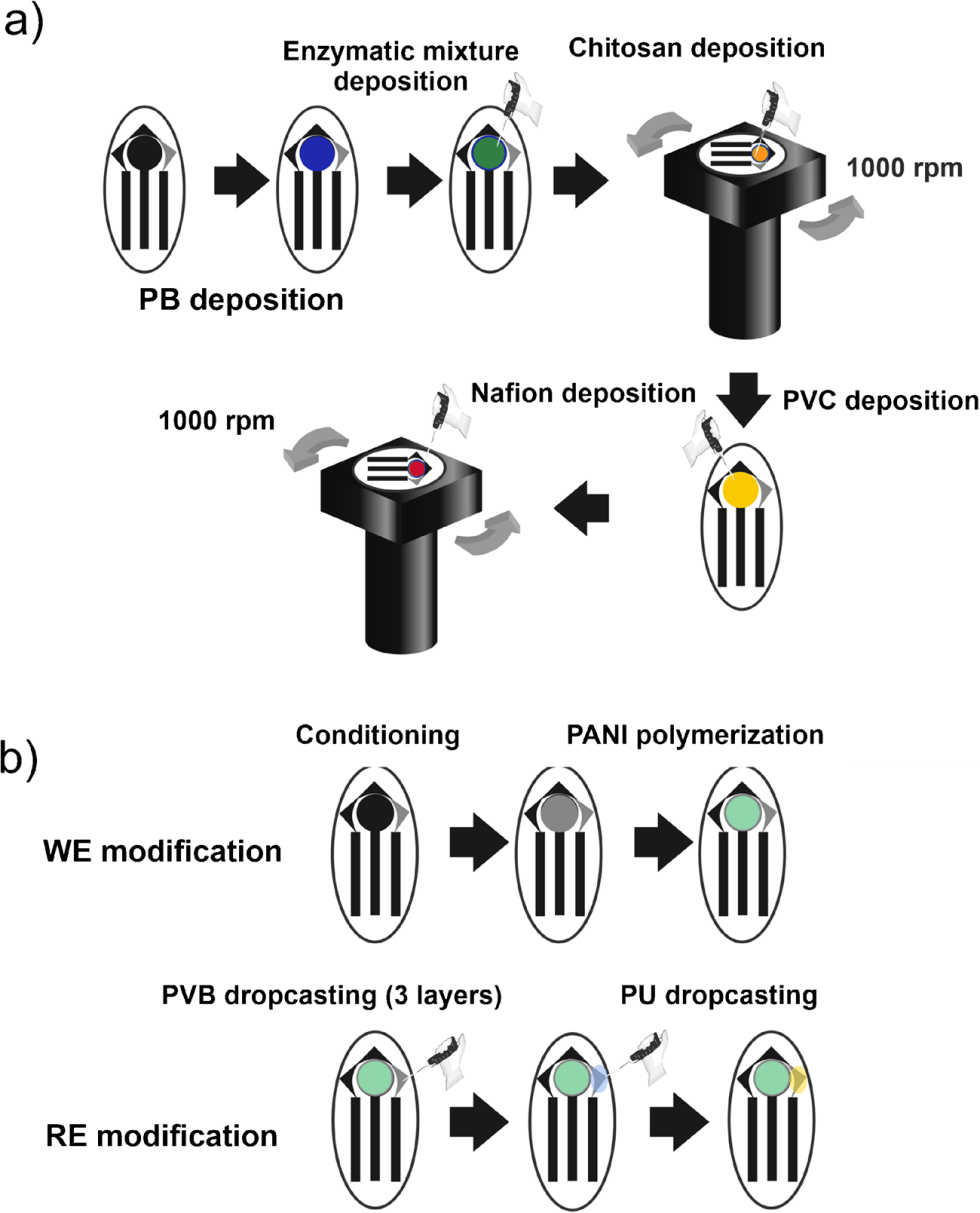
Fabrication scheme of the (bio)sensors developed. **a** Fabrication of SCPC-PB-LOxAuNPs-Chit-PVC-Nafion® biosensor. **b** Fabrication of PANI-based pH probe

A scheme of the fabrication of the (bio)sensors developed is shown in Fig. 2, and their fabrication was carried out as follows.

Regarding the lactate biosensors (Fig. 2a), SCPCs were firstly conditioned in PBS 0.1 M pH 7.4, at 1.8 V during 180 s. Afterwards, they were modified with Prussian blue (PB) by using a similar procedure to one found in literature [24]. Briefly, 12 µL of a solution containing 0.1 M FeCl_3_ and 0.1 M KCl in 0.01 M HCl was drop casted onto the surface of the carbon working electrode (WE). After then, 12 µL of a solution containing 0.1 M K_3_Fe(CN_6_) and 0.1 M KCl in 0.01 M HCl was also drop casted onto the WE. After 20 min, the final mixture was removed from the WE, and it was rinsed by using 40 µL of 0.01 M HCl and 40 µL of nanopure water. The resulting modified electrodes were annealed at 100 °C for 1 h in an oven. Once the electrodes were dried at room temperature, 20 µL of a solution containing 100 U of LOx in 260 µL of a AuNPs colloidal solution previously synthesized using a procedure already reported [25] was deposited by drop casting and left it to dry for 2 h under vacuum at − 200 mbar. Before making the enzymatic/AuNPs mixture, the synthesized AuNPs were characterized by UV–vis spectrophotometry (Fig S1a) and dynamic light scattering procedures (Fig S1b). The former studies confirmed the presence of AuNPs due to the plasmonic resonance surface found at 536 nm. The latter indicated a narrow size population distribution with a weighted mean of 3.2 ± 1 nm.

The integration of the diffusion limiting layers was carried out in the next steps. First, 30 µL of a solution of 1% wt chitosan in 0.1 M acetic acid at pH 4.5 was placed on the WE and spin coated by using the homemade rotor instrument at 1000 rpm under the hood for 1.5 h. Once the drying step was completed, 4.2 µL of a solution containing 1% wt PVC in tetrahydrofuran was drop casted onto WE and left to dry at room temperature. This step was repeated 3 times in total. Finally, 11.5 µL of a solution containing 1% wt Nafion® in ethanol at pH 7.5 was placed onto WE and spin coated at 1000 rpm under the hood until it was dried. The resulting biosensors were stored dried at 4 °C ready to use.

Once biosensors are prepared and before their storage at 4 °C, the sensitivities provided for the lactate determination ranging from 0.5 to 20 mM were obtained. Before performing the second use of the biosensor selected, other calibration plot under same conditions was recorded, and after then, sensitivity value obtained was compared with the initial one; the biosensor was discarded if the difference between both is higher than 20%. For the prototype sensors reported in this paper, their discard procedure is based on their disposal to the adequate recipient for biological hazardous materials and its subsequent incineration. For further mass-produced and miniaturized devices, the electronic part will be discarded accordingly, while the part in contact with the human body (i.e., microneedles) will be disposed in the same way as biological hazardous materials.

The fabrication of the pH sensor was carried out following the procedure shown in Fig. 2b. A PANI layer was electrodeposited onto the working electrode surface of the screen-printed sensor by following the procedure described elsewhere [23]. Afterwards, the reference electrode was modified by casting 2.5 µL of a 7.5% (%w/v) PVB and 0.85 mM NaCl solution dispersed in methanol and left to dry for 30 min. This procedure was repeated twice, resulting in three layers of PVB deposited on RE. Later, 2.5 µL of 3% polyurethane solution dispersed in tetrahydrofuran was deposited on RE by drop casting, leaving it to dry for 1 h.

### Electrochemical measurements performed in batch mode

The preliminary electrochemical studies performed with the biosensor were developed in batch mode. The LA determination was accomplished with the final configuration of the biosensor in a range from 0.5 to 20 mM (PBS 0.1 M, pH 7.4) by using amperometry at 0.05 V. The interference study was accomplished using relevant species, such as ascorbic acid (AA), uric acid (UA), dopamine (DA), and glucose.

Regarding the pH sensor, the measurements were accomplished by potentiometry using PBS solutions ranging from 6 to 8, adjusted with KOH. Highly likely interferents such NaCl, CaCl_2_, KCl, MgCl_2_, and glucose were employed in the interference study.

### Flow injection analysis assays

The analytical performance of the (bio)sensors was also assessed in flow regime at 0.05 V. A constant flow rate of 0.27 mL min^−1^ was kept for the electrochemical assays. 0.1 M PBS at pH 7.4 was selected as carrier solution and, once a stable signal was obtained (about 200 s), 680 µL of LA stock solution ranging from 0.5 to 20 mM was injected with the assistance of a six-way valve.

Regarding the real sample analysis, the calibration in presence of stock LA solutions ranging from 0.5 to 20 mM with a fresh biosensor was performed. After then, independent injections of 200 µL of the corresponding serum samples were carried out, recording every signal obtained after each injection. Finally, the LA concentration values were estimated by using the previous calibration plot. In total, nine human serum samples were collected from healthy volunteers (ca. 2 mL each). This sample volume exceeds the required amount measured with the electrochemical probe under this regime. The remaining sample was stored for future studies.

Likewise, pH measurements were performed using a PANI-based electrochemical sensor in a flow regime, using a flow rate of 1.07 mL min^−1^. PBS solutions with pH values ranging from 6 to 8 were employed, cleaning the system with nanopure water between each buffer solution. Concerning the real sample analysis, nine human plasma samples were analyzed in flow regime using the electrochemical probe.

## Results and discussion

### Selection of multipolymeric membrane as diffusion layer

With the aim to avoid enzymatic saturation at high LA levels, the integration of a diffusion limiting layer is proposed. The evaluation of the linear range provided with different feasible biocompatible polymers was carried out. As abovementioned in the “Introduction” section, thicker, low porous, and hydrophobic layers conduct to higher improvements in the linear range response due to higher hindrance for the analyte to reach the sensor. It is noteworthy to mention that the polymer coatings over Pt substrates were performed by using drop casting method. Furthermore, same enzyme loadings were deposited in all cases (see supplementary information for details). Chitosan, cellulose acetate, polyurethane (PU), and polyvinyl chloride (PVC) were selected as biocompatible polymers. The results can be observed in Fig. S2 found in the supplementary material. Interestingly, cellulose acetate reaches about 5 mM of limit of detection but high dispersion in the results was found (Fig. S2a). This fact can be explained due to the instability of the cellulose layer in long-term measurement (> 1000 s). Eventually, in this scenario, the layer detaches spontaneously, but surely, the process occurs gradually altering the measurement. Remarkably, biosensor modified only with PU provided a mediocre linear range response (3 mM), but it is increased towards 8 mM when a chitosan layer is placed before the PU layer (Fig. S2b). A likely explanation resides in the enzyme environment found in the biosensor. The suitability of chitosan to entrap enzyme retaining their activity has been remarked by many authors [26]. In this regard, it was stated that this enzyme immobilization role is mainly ascribed to the mild acidic pH as well as the biocompatible ramifications of this polymer [27]. Thus, two contributions could be highlighted: an appropriate layer of chitosan may preserve the enzyme and the additional PU layer acts as a more classical diffusion limiting layer. This phenomenon is also observed when PVC is employed. Single chitosan layer does not lead to the required range (2 mM), but by using 1% of PVC it is possible to reach 10 mM (Fig. S2c). Even though this is not enough for real applications, the improvement is notorious. Remarkably, the combination of chitosan and PVC brings also a range of 10 mM, but also higher sensitivity (estimated as the slope of the calibration plot). Thus, a dual layer of chitosan 1% and PVC 1% is the configuration selected for further studies.

### Spin coating procedure for SCPC-PB-LOxAuNPs-Chit-PVC-Nafion® lactate biosensor

In this work, we aim to exploit spin coating approach to improve the features of the diffusion limiting layer. It should be noted that Prussian blue was employed as redox mediator, since the hydrogen peroxide reduction is carried out at lower potential (around 0 V), avoiding interferences with other species and, therefore, enhancing the selectivity of the resulting biosensor. After performing the deposition of PB layer, gold nanoparticles (AuNPs) synthesized by using a green ultrasound-assisted approach previously published [25] were included in the enzymatic mixture pursuing some analytical advantages to the resulting biosensor, such as electrocatalytic effects and better enzyme immobilization. Once AuNPs were characterized, the enzymatic mixture was drop casted on top of the already set PB layer. After drying, the diffusion layers were included by using spin coating procedure, as explained in the experimental section.

Spin coating was carried out by using a homemade rotor and some 3D printed tools to adapt the screen-printed electrodes (Figure S3). Several assays were carried out to fix a certain speed and amount of solution. Visual inspection of the resulting coatings in the biosensors conducted to a speed of 1000 rpm in all cases. Speed values higher than 1500 rpm causes loss of the amount deposited, leading to no homogeneous layer. On the other hand, lower speeds than 500 rpm did not offer different surface features in the membranes in comparison with the drop casted one (data not shown). The volume deposited is another relevant parameter. In the case of chitosan, 30 µL was selected as optimum amount to cover the WE and avoid projections during the spin coating at the same time. In contrast, PVC was not deposited by using spin coating procedure due to the volatility of its solvent, tetrahydrofuran, and hence, no reproducibility in the deposition process could be accomplished. Finally, a Nafion® layer is also placed onto the electrode surface as anionic barrier, as it was stated in the “Introduction” section. Contrarily to PVC, Nafion® was dissolved in ethanol, which allows a deposition carried out by spin coating. The optimal volume selected was 11.5 µL, based on the same reasons previously exposed for chitosan. The surfaces of the prepared biosensors were examined by using atomic force microscopy (AFM). An analogue biosensor prepared using only drop casting method was also characterized for comparison purposes.

A porous surface can be appreciated in the membrane deposited by drop casting procedure (Figure S4a), with a mean roughness of 196.1 nm estimated by using Gwyddion software. On the other hand, the spin coated resulting layer (Figure S4c) possesses less porosity, with a mean roughness of 33.85 nm. This can be translated into a more solid and cohesive membrane that will aid to expand the linear response range of the resulting device. Additionally, the visual inspection from optical microscopy is exposed in Fig. S5 and reflects a highly rough and porous layer in the case of the drop casted layer (Fig. S5a). On the contrary, spin coated layer (Fig. S5b) presented a more cohesive and thicker layer with less porosity. This is corroborated by the AFM characterization previously exposed (Figure S4).

### Analytical performance of the SCPC-PB-LoxAuNPs-Chit-PVC-Nafion® lactate biosensor in batch regime

**Fig. 3:**
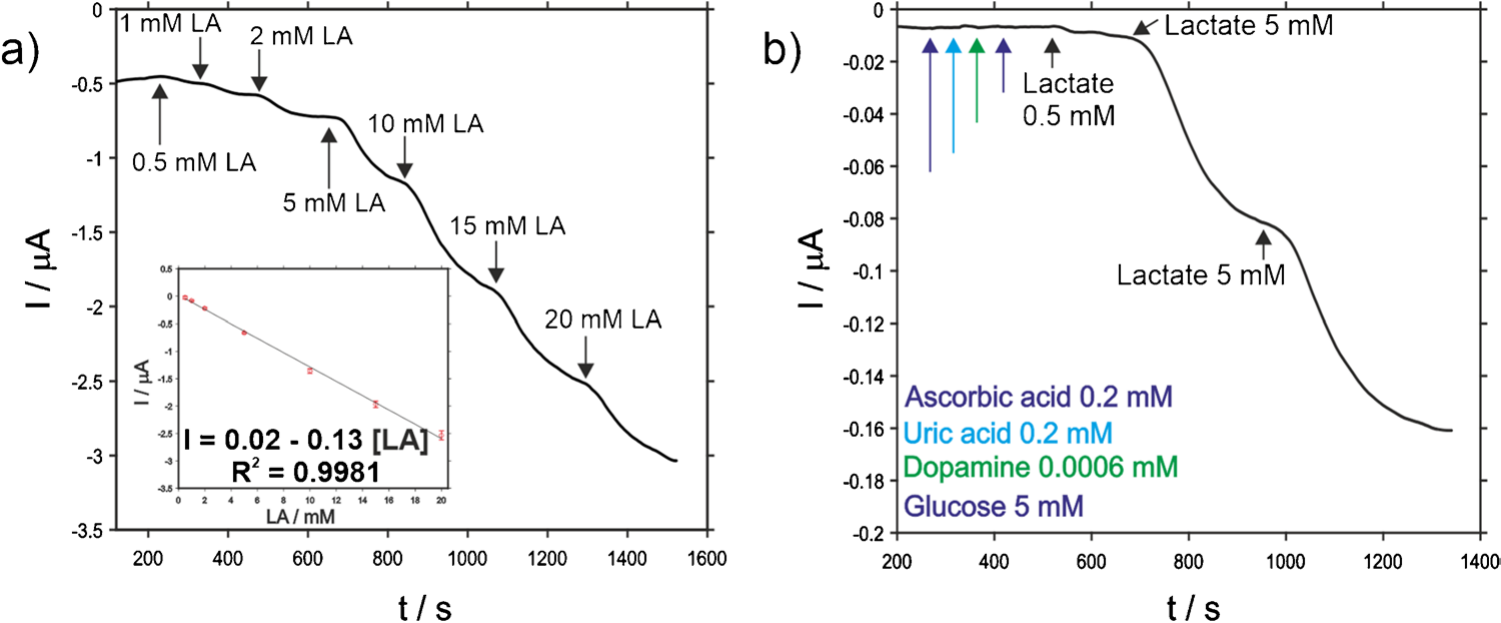
Analytical performance of the SCPC-PB-LOxAuNPs-Chit-PVC-Nafion® lactate biosensor in batch regime in PBS 0.1 M pH 7.4. **a** Chonoamperogram (*E* = 0.05 V) corresponding to the calibration plot of LA ranging from 0.5 to 20 mM; (inset: lactate calibration plot). **b** Interferent assay performed by using ascorbic acid (AA), uric acid (UA), dopamine (DA), and glucose (GL) as interfering species at 0.2, 0.2, 0.006, and 5 mM, respectively, prior to the addition of 0.5, 5, and 5 mM of LA

The amperometric sensing of LA was carried out using the developed biosensor in batch regime. Before analysis, the resulting biosensor was characterized by cyclic voltammetry in free analyte solution (Fig. S6), revealing an anodic peak around 0.05 V and a cathodic one around − 0.08 V, frequently ascribed to the presence of PB in literature [24]. The peak shifts and current decrease can be explained by the inclusion of enzyme and less conductive materials to the electrode. It is expected that the presence of hydrogen peroxide will lead to the oxidation of the reduced form of Prussian blue, and thus, the application of a potential around 0.05 V will drive again to a cathodic current increase. Concerning the calibrations, lactate monitoring was successfully performed ranging from 0.5 to 20 mM of LA (Fig. 3a).

As it was stated before, the current signal decreased after each addition of LA, which can be ascribed to the catalytic reduction of the hydrogen peroxide derived from the enzymatic reaction of lactate. The results revealed a linear relationship between the current and the concentration at the concentration range tested (*R*^2^ = 0.9981), as well as a low dispersion of the results. In fact, repeatability and reproducibility estimated (*n* = 3) were 0.84 and 2.66%, respectively, which indicates the robustness of the preparation methodology. Regarding other analytical parameters, the sensitivity, calculated as the slope of the calibration plot, and limit of detection, estimated as three times the standard deviation of the blank, were 0.129 ± 0.001 µA mM^−1^ cm^−2^ and 0.18 mM, respectively.

The analytical parameters were compared with those found for other amperometric biosensors based on LOx, shown in Table 1.

**Table 1:** Comparison of analytical performance for LA amperometric biosensors found in literature

Sensor	Technique	Sensitivity (µA/mM cm^2^)	Limit of detection (µM)	Linear range (mM)	Ref
SPCE-PB-LOx + GO-Chit	FIA	0.39	0.028	1–50	[14]
SPE/Graphene/PB/PVA-SbQ-LOx	FIA	1.64	15*	0.25–5	[28]
Pt/rGO/CNT/AuNPs/LOx	Chronoamp	35.3	2.3	0.005–100	[8]
SPE-ERGO-PAH-AuNPs/LDH-GA	Chronoamp	1.08	1	0–3	[29]
Au-PB-Chit/CNTs-LOx-Chit/CNts	Chronoamp	0.220	-	0–30	[18]
CE-fSWCNTs/Chit-PBNPs/Chit LOx	Chronoamp	-	200	1–25	[30]
G-paper/MoS_2_/Cu/LOx	Chronoamp	83	0.5	0.005–18	[31]
N-graphene/Ti_**3**_C_**2**_Tx/PB/LOx	Chronoamp	21.6	2.3	0–20	[32]
Ti-PTFE/PPy-MWCNTs	Chronoamp	2.9	51	1–15	[11]
SPCE-NiCo (layered double hydroxide)	Chronoamp	84	400	2–26	[33]
SPCE-PB-LOxAuNPs-Chit-PVC-Naf	Chronoamp	0.13	180	0.5–20	This work
SPCE-PB-LOxAuNPs-Chit-PVC-Naf	FIA	0.19	120	0.5–20	This work

*SPCE* screen-printed carbon electrode, *PB* Prussian blue, *LOx* lactate oxidase, *GO* graphene oxide, *Chit* chitosan, *SPE* screen-printed electrode, *PVA-SbQ* poly(vinyl alcohol) bearing styrylpyridinium groups, *rGO* reduced graphene, *CNT* carbon nanotubes, *AuNP* gold nanoparticles, *ERGO* electrochemical reduced graphene oxide, *PAH* poly(allylamine) hydrochloride, *LDH* lactate dehydrogenase, *GA* glutaraldehyde, *fSWCNTs* functionalized single-walled carbon nanotubes, *PBNPs* Prussian blue nanoparticles, *G-paper* graphene paper, *N-graphene* N-doped graphene, *PTFE* polytetrafluoroethylene, *PPy* polypyrrole, *MWCNTs* multiwalled carbon nanotubes, *FIA* flow injection analysis, *Chronoamp* chronoamperometry.

*Estimated from the data shown.

The sensitivity provided with the device is at the same level as those obtained with biosensors exposed in the previous table. Importantly, diffusion limiting layer aids to the expansion of the linear response range, but it also has influence in the resulting sensitivity of the sensors. It is noteworthy to mention that this limiting layer was placed in a fast, reproducible, and easy manner by using spin coating procedure; as far as we are concerned, this is the very first time this procedure is reported in literature for diffusion limiting layer construction. For this reason, it is assumed that sensors which includes a diffusion limiting layer cannot match the sensitivity from their analogous uncoated sensors [14]. This decrease in the sensitivity is not a critical concern for real-life scenarios, since blood lactate concentration in healthy and unhealthy situations is around 0.5–25 mM [34]. Thus, the concentration range reached by the biosensor is suitable for the detection of critical situations of lactic acidosis and may allow monitoring of real-life patients.

Other relevant parameters should be considered. The integration of a PB membrane as redox mediator in the device, only employed in some examples reported in the previous table, leads to a high selectivity for the LA determination due to the catalytic reduction of the hydrogen peroxide, the subproduct of the enzymatic reaction of LA. Regarding the response time, an average value of 160 s was calculated as the time needed to reach the 95% of the steady-state signal. This value is dependent on the concentration of LA, being lower at small concentration values and higher near the maximum limit. This concentration dependence can be explained by the diffusion limiting layer placed, which hinders the transport of the analyte into the enzymatic layer. However, a delay in the response less than 3 min is not considered meaningful for long-term measurements, required in real-time applications. Finally, the low manufacturing cost of the device can be stated (ca. 25 € including the enzyme cost), in comparison with the cost required for the manufacturing of other sensors based on expensive nanomaterials, such as graphene or single-walled carbon nanotubes. These features favor the possibility for mass production and further miniaturization, which may fulfil the requirements in field clinical diagnosis. In addition, the previous cost is expected to be significantly reduced when commercialized.

The selectivity of the biosensor developed was assessed by using common species found in the organism with high electroactivity such as ascorbic acid, uric acid, dopamine, and glucose. Particularly, the concentrations assayed were 0.2, 0.2, 0.006, and 5 mM, respectively, which are higher than those employed in other pieces of research reported in literature [16]. As it can be noticed in Fig. 3b, the baseline is not disrupted by the presence of these molecules at the assessed concentrations, while current changes were recorded after the corresponding additions of LA aliquots. This outstanding selectivity may be explained by several factors: (i) the decrease of the working potential due to PB mediator; (ii) Nafion® layer drives away anionic interferent species; and (iii) a solid spin coated diffusion limiting layer greatly hinders the diffusion of the analyte as well as the interferent species.

### Analytical performance of the SCPC-PB-LOxAuNPs-Chit-PVC-Nafion® lactate biosensor and the pH PANI-based probe in flow regime

**Fig. 4:**
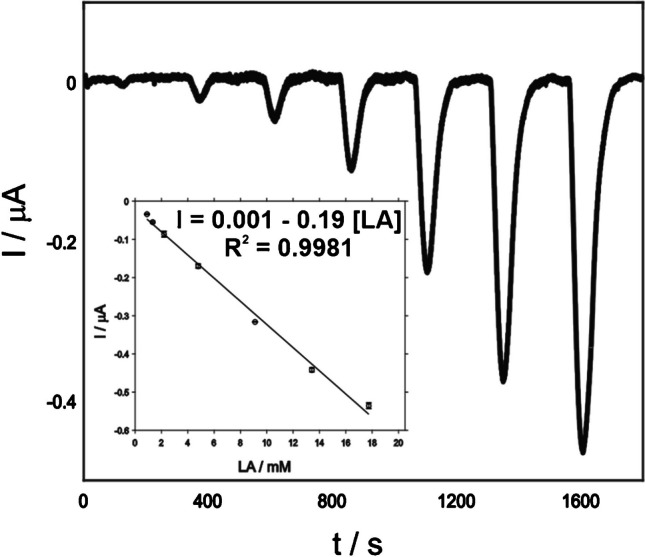
Chonoamperogram recorded in presence of LA ranging from 0.5 to 20 mM in 0.1 M PBS at pH 7.4 with the biosensor developed in flow regime at 0.05 V. The inset shows the lactate calibration plot

Flow analysis approach has been employed to mimic the real environment of the final application of the sensors developed. Continuous monitoring is the final goal of this kind of devices, providing real time and meaningful information concerning the patients in healthcare ambit. Particularly, lactic acidosis careful assessment may impact very positively in the survival ratio. Therefore, the developed lactate biosensor was evaluated in flow regime. Regarding the lactate biosensor performance, the results are exposed in Fig. 4.

A calibration plot was recorded by using LA concentration in a similar range than the one used in batch regime (0.5 to 20 mM). The sensitivity and the LOD are 0.19 µA mM^−1^ cm^−2^ and 0.12 mM, respectively. These values are similar to those found in batch mode. Importantly, the linearity is still excellent (*R*^2^ = 0.9981), and thus, the biosensor is suitable for the detection of critical situations of lactic acidosis. In addition, the baseline is stable for long periods of time (see Fig. S7), and a repeatability of 4.2% was found. This repeatability was estimated as the RSD (%) of the slopes derived from three calibration plots recorded with the same biosensor.

Regarding the analytical performance of the electrochemical device, the storage time should be considered before use, since the sensitivity values for lactate determination provided decreases after 1 month of storage. As it was exposed in the experimental section, once the sensitivity provided with the biosensors decreased around 20% in comparison with the initial value, they were discarded to obtain reliable results.

**Fig. 5:**
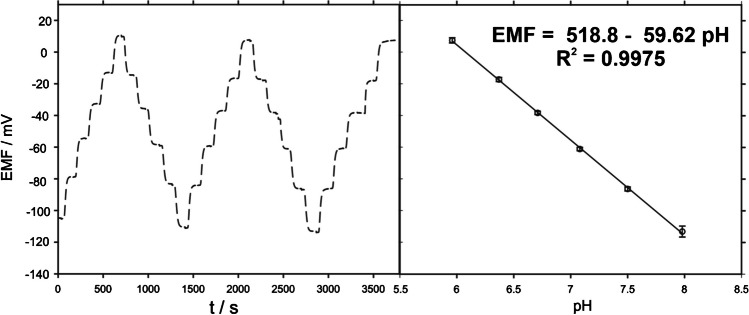
Dynamic response and average calibration graph for the carry-over test (five consecutive calibrations by alternatively increasing and decreasing the pH in buffer solutions) performed with the PANI-prepared pH sensor

As it was discussed in the “Introduction” section, the electrochemical results obtained with the biosensor could be assisted using a pH probe for lactic acidosis monitoring. In this sense, a common PANI**-**based electrochemical sensor was developed as an electrochemical probe. An evaluation via cyclic voltammetry was performed to check the presence of the conducting polymer after the electrodeposition, revealing the corresponding peaks ascribed to the oxidation–reduction process of the leucoemeraldine-emeraldine and emeraldine-pernigraniline phases (Fig. S8). Particularly, the peaks found are slightly shifted due to the layer thickness, but their existence suggested a successful electrodeposition of PANI on the surface of the SCPC. Concerning the analytical performance, several consecutive calibrations were performed in a pH range from 6 to 8 pH (Fig. 5).

As it can be appreciated in Fig. 5, EMF values decrease with the increase of the pH, which can be ascribed to the redox equilibrium between protons and PANI phase transitions [35, 36]. In this sense, the conductivity of PANI chain is associated with the reversible transition between the emeraldine salt and emeraldine base, and thus, the higher deprotonation degree of PANI leads to a lower potential output signal.

Concerning the analytical features, a sensitivity of 59.62 mV and a *R*^2^ of 0.9975 were found, demonstrating the capability of the pH sensor in the selected range. On the other hand, the drift of the sensor was studied for more than 10 h (Fig. S9), obtaining an estimated value of 0.6 mV h^−1^, which can be used as a correction factor to improve the accuracy in long-term measurements. Additionally, the pseudoreference electrode from the commercial SCPC was modified by using a membrane based on polyvinyl butyral (PVB) and PU to provide enhanced stability, previously reported in literature with excellent results [37]. The analytical performance of the modified reference electrode has been evaluated in comparison with the classical double junction commercial reference electrode (Fig. S10). No meaningful differences between the slopes recorded with both were found.

The selectivity coefficients of different feasible interfering species, either ions or organic compounds, were calculated by using the separate solution method (SSM) [38] and Debye-Hückel approximation for activity estimations [39] (Table S1). Briefly, these coefficients are an indicative of the affinity of the sensor towards the analyte in presence of the interfering species. Besides, the smaller the value, the greater the sensor preference for the target analyte. All the coefficient values estimated are negative, which can be translated into an adequate selectivity towards the studied species at the activities assayed (log(a) from − 5 to − 1).

For all these reasons, we consider that the SCPC-PB-LOxAuNPs-Chit-PVC-Nafion® lactate biosensor and the pH PANI-based probe displayed suitable analytical features for the direct measure of biological samples, such as human serum or even blood.

### Real sample analysis

**Fig. 6:**
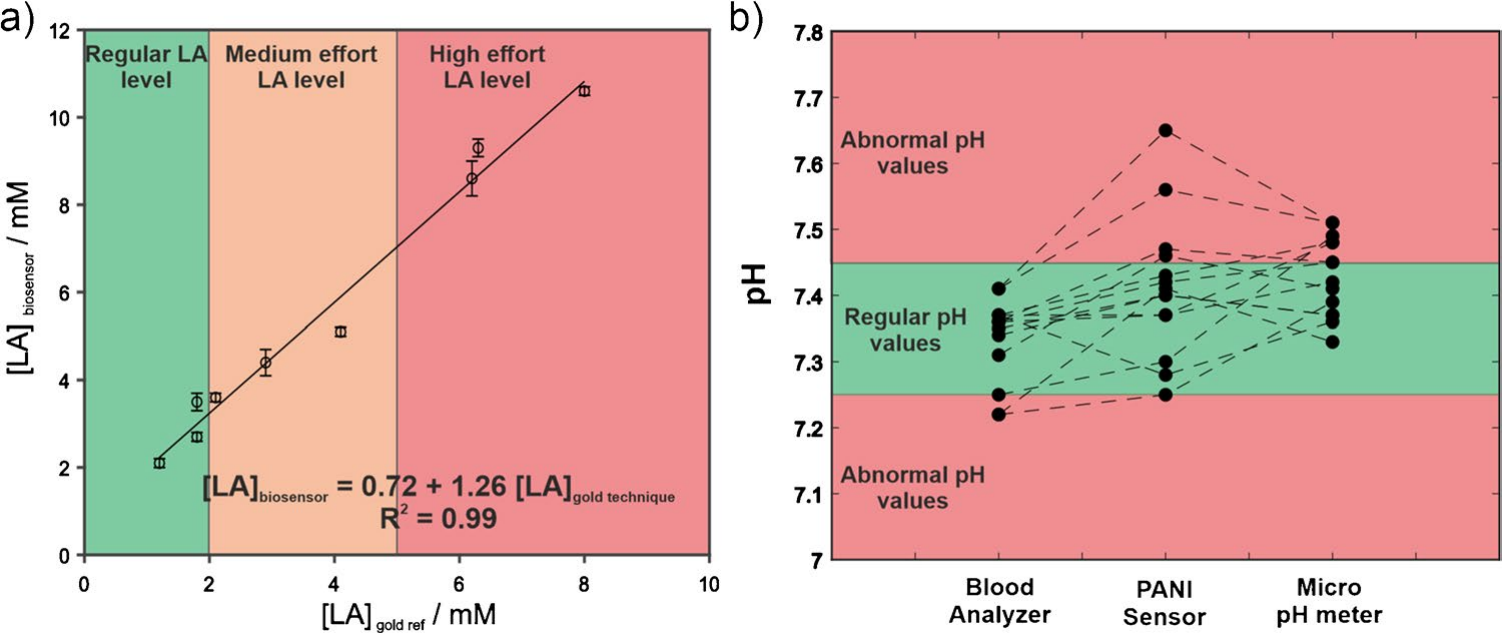
**a** Correlation between the concentration values of LA determined with the blood analyzer in serum samples and those determined with the biosensor developed. **b** Results of pH obtained by the blood analyzer, the PANI pH sensor developed, and a micro pH-meter

The developed biosensor was employed for the flow analysis of LA in raw serum samples collected from different volunteers; some of them previously subjected to physical exercise to increase their LA levels. Before analysis, the serum LA concentration for each sample was measured by means of the gold technique. Remarkably, blood gas analyzer has been already used by other authors in literature [40]. Nevertheless, a positive linear correlation of both variables can be found, as it can be appreciated in Fig. 6a.

The equation of the linear regression curve is shown below.

$${[LA]}_{biosensor}=1.26\times {[LA]}_{gold technique}+0.72$$

where [LA]_biosensor_ is the concentration found using the biosensor developed and [LA]_gold technique_ is the corresponding one found by means of the blood analyzer.

The correlation coefficient of the statistical model is 0.988, indicative of a strong correlation between both parameters. To examine the presence of outliers, the studentized residual values calculated were plotted in Figure S11. Based on these results, no outliers could be stated. Additionally, no systematic errors are present as no trend in the residual values can be found.

There is, indeed, slight differences between both approaches; however, discrepancies are also usually found in sweat samples [41], being accepted in some cases a deviation of 20% without applying any correction factor [42]. In our case, the real sample matrix is more complex and, therefore, some differences are expected. Besides, the excellent correlation obtained in our work overpasses other studies carried out with blood lactate found in literature [43]. Based on these results and according to the strong correlation between both parameters, LA concentration in raw human blood serum could be estimated accurately after applying the corresponding correction factor by using the equation previously described.

All the serum samples were randomly analyzed using the same biosensor, indicating no memory effects and negligible passivation phenomena. Once the buffer solution flow is restored, the signal increases until the baseline is recovered. This means that the baseline was reached by the simply flowing of the buffer solution around 200 s, and after then, the biosensor is ready to analyze the following serum sample. Based on this result, the reusability of the biosensor device is highlighted.

Regarding the pH determination, human plasma samples collected from diverse volunteers were in situ analyzed with the pH PANI probe after collection with the aim to avoid significant pH changes ascribed to the storage process. Some of the volunteers were subjected to physical exercise to decrease their pH levels. Figure 6b exposes the experimental results obtained with the electrochemical sensor as well as the values obtained with commercial sensors either, the one located within the blood-gas analyzer and a regular pH-micrometer. According to the results reported, the PANI probe is able to detect pH in a suitable range, with regular values close to 7.35, and the exercise one close to 7.22. Remarkably, no statistical significances using *T*-test (95% of confidence interval) between the pH values obtained using the PANI probe in comparison with those obtained with commercial sensors were found, demonstrating its utility in the pH monitoring. Interestingly, there are differences between the reference techniques, pointing out that our values are more in agreement with the regular pH-meter, highly contrasted. One possible explanation might be the not enough accuracy of the blood gas analyzer used in surgery rooms. This also could explain the slight differences in the previous LA scenario. Anyway, much deeper studies need to be done in this sense in further research.

## Conclusions and future works

In this work, a spin coated multipolymeric layer LOx-based biosensor was successfully developed for the electrochemical analysis of LA. Suitable figures of merits for LA determination were displayed from 0.5 to 20 mM, wide concentration range for analytical monitoring in relaxed mode and under exercise. Satisfactory results with the biosensor in flow regime were also provided in terms of sensitivity and selectivity, demonstrating its feasibility for electroanalytical purposes in continuous. The concentration of LA in raw serum samples collected from volunteers in relaxed mode and after physical exercise could be also successfully estimated. On the other hand, LA analyses were supported by the pH measurements using an electrochemical method, displaying excellent performance in the pH determination of stock solutions and untreated plasma samples, covering pH values ranging from 7.22 to 7.40.

Based on the results exposed in this article, the biosensor constitutes a promising tool for the lactic acidosis monitoring using a fast, robust, portable, and reliable method. After its further miniaturization, the device could be employed for the assessment of LA as relevant biomarker in sport medicine. Furthermore, the early diagnosis of severe complications derived from lactic acidosis in risk patients, such as hypoxia, could be avoided, or even minimized.

With the aim to aid early and meaningful diagnosis in several scenarios, the development of a tandem LA and pH electrochemical device coupled with microneedles for the simultaneous monitoring of both parameters in biofluids is proposed in future works. Besides, further studies will also include the employment of another reliable and typical reference analytical technique to validate the final device and to minimize the error rate.

## Supplementary Information

Below is the link to the electronic supplementary material.

Supplementary file1 (DOCX 167685 KB)

## Data Availability

No datasets were generated or analysed during the current study.
