# Supplementary material for: New spin coated multilayer lactate biosensor for acidosis monitoring in continuous flow assisted with an electrochemical pH probe
Source: Mikrochim Acta. 2024 Aug 9;191(9):526. doi: 10.1007/s00604-024-06602-y (PMC11315777; doi:10.1007/s00604-024-06602-y)
Supplement: Supplementary file 1 — Supplementary file1 (DOCX 167685 KB) [file 604_2024_6602_MOESM1_ESM.docx]

**SUPPLEMENTARY MATERIAL**

New Spin Coated Multilayer Lactate Biosensor For Acidosis Monitoring In Continuous Flow Assisted With An Electrochemical pH Probe.

Juan José García-Guzmán^a^, José Manuel Jiménez Heras^a^, David López-Iglesias^a^, Rafael Jesús González-Álvarez^a^, Laura Cubillana-Aguilera^b,*^, Carmen González Macías^c^, Juan Jesús Fernández Alba^c,*^, José María Palacios-Santander^b^

a) Instituto de Investigación e Innovación Biomédica de Cádiz (INiBICA), Hospital Universitario ‘Puerta del Mar’, Universidad de Cádiz, 11009 Cádiz, Spain. b) Institute of Research on Electron Microscopy and Materials (IMEYMAT), Department of Analytical Chemistry, Faculty of Sciences, Campus de Excelencia Internacional del Mar (CEIMAR), Universidad de Cádiz, República Saharaui, S/N. 11510 Puerto Real, Cádiz, Spain. c) Departamento de Obstetricia y Ginecología. Hospital Universitario de Puerto Real, 11510 Puerto Real, Cádiz, Spain.

*Correspondence: [laura.cubillana@uca.es](mailto:laura.cubillana@uca.es); [jjesus.fernandez@uca.es](mailto:jjesus.fernandez@uca.es)

**Figures**

Fig. S1. Characterization of colloidal gold nanoparticles solution synthesized using high-power ultrasound: a) Spectrum recorded using UV-Vis Spectroscopy and b) Distribution size using Dynamic Light Scattering.

Fig S2. Calibration plots of different Pt-LOx based biosensors during lactate determination in PBS 0.1 M pH 7.4, by using different polymers to fabricate the diffusion limiting layer: a) acetate cellulose, b) polyurethane and mixture chitosan/polyurethane, c) polyvinyl chloride and mixture chitosan/polyurethane.

Fig S3. a) Representation of tools designed to assist the spin-coating procedure printed by additive manufacturing; b) real set up deployment of the screen-printed electrode integrated in the homemade-rotor.

a)

b)

Figure S4. 3D AFM images of SCPC-PB-LoxAuNPs-Chit-PVC-Nafion® biosensors performed by drop casting procedure at 10 (a) and 25 (b) µm^2^ and prepared by spin coating procedure at 10 (c) and 25 (d) µm^2^.

Fig S5. Visual inspection via optical microscopy of the SCPC-PB-LOxAuNPs-Chit-PVC-Nafion lactate biosensor prepared: a) by dropcasting procedure and b) by spin-coating procedure.

Fig S6. Cyclic voltammograms recorded with different Prussian Blue-based electrochemical sensors in 0.1 M PBS at pH 7.4

Fig. S7. Amperogram recorded with the developed biosensor in 0.1 M PBS for 4000 s.

Fig. S8. Cyclic voltammogram recorded with the pH polyaniline probe in 0.1 M HCl solution.

Fig. S9. Long-term response evaluation of pH Polyaniline probe.

Fig. S10. Calibration curve recorded with the homemade reference electrode and a commercial one using buffer solutions at different pH values.

Fig. S11. Studentized residual values for the biosensor validation.

Table S1. Selectivity values calculated by the SSM method for the PANI pH probe.

| Interferents (J) | Selectivity coefficient (K_IJ_) |
| --- | --- |
| Na^+^ | -6.21 |
| Ca^2+^ | -9.03 |
| Mg^2+^ | -11.67 |
| Glucose | -4.19 |
| Urea | -5.01 |
